# Supplementary material for: Comparing the test performance of dried-blood-spot and plasma HIV recent infection testing samples in a nationally scaled sex worker programme in Zimbabwe
Source: PLOS Glob Public Health. 2025 Nov 5;5(11):e0003791. doi: 10.1371/journal.pgph.0003791 (PMC12588490; doi:10.1371/journal.pgph.0003791)
Supplement: Attachment — Submitted filename: Review Comments _PGPH-D-24-02067_JullyOkonji.docx [file pgph.0003791.s001.docx]

Reviewers Comments

T**itle:** Comparing the test performance of dried-bloodspot and plasma HIV 2 recent infection testing samples in a nationally scaled sex worker programme 3 in Zimbabwe

Manuscript N0. PGPH-D-24-02067

Generally, the manuscript is well-written and organized

The study presents an important topic on recency testing and evaluates the use of DBS vs. Plasma for recency testing. The authors have described the study well in the introduction and its objective.

Line 65-66 (**‘HIV 65 testing coverage has increased substantially and those attending services are likely to be representative of female sex workers (FSW) in Zimbabwe)**, the author’s statement highlighted in bold suggests that they are unsure of the population used in the study. Did you confirm that the enrolled participants were sex workers or were they persons seeking services from the mentioned clinics?

The authors have used the Viral load test, HIV Lag, Avidity and ELISA test in determining the thresholds for either recent or long-term infection. However, the authors did not mention which ELISA kit was used, or the manufacturer’s name, similar to the HIV LAg Avidity test used in this study.

The authors have not mentioned the sample input volume used in the testing for HIV Viral load, ELISA, and HIV LAg Avidity.

Could it be that the threshold used for HIV viral load testing using the DBS matrix contributed to the over classification of more recent infections compared to plasma Viral load?

**Suggestion for your consideration:**

See line 105-Can the authors consider presenting the data on the 58 recent infections in a 2by-2 table and have the sensitivity and specificity determined for each sample matrix?

Could the results have been different if DBS were accorded similar storage treatment as plasma from collection to storage instead of having them stored at room temperature for 14 days ? Could this have been a limitation in the design of this study?

**Recommendation:** Areas that need review by the authors are considered minor.
