# Supplementary material for: Comparing the test performance of dried-blood-spot and plasma HIV recent infection testing samples in a nationally scaled sex worker programme in Zimbabwe
Source: PLOS Glob Public Health. 2025 Nov 5;5(11):e0003791. doi: 10.1371/journal.pgph.0003791 (PMC12588490; doi:10.1371/journal.pgph.0003791)
Supplement: Attachment — Submitted filename: Rice et al_DBS_Response to reviewers.docx [file pgph.0003791.s003.docx]

16^th^ January 2025

Dear Editors,

**Response to reviewer comments on a submitted research article – Comparing the test performance of dried-blood-spot and plasma HIV recent infection testing samples in a nationally scaled sex worker programme in Zimbabwe(PGPH-D-24-02067).**

We wish to thank the editors of PLOS Global Public Health for considering our submission for publication. We also wish to thank the reviewer for their support and guidance. Below we provide detail of our response to each of the editorial and reviewer comments.

**Editorial comments**

**1. Please include a complete copy of PLOS’ questionnaire on inclusivity in global research in your revised manuscript……..Please upload a completed version of your questionnaire as Supporting Information when you resubmit your manuscript.**

As requested, we have completed the questionnaire on inclusivity and we have uploaded this with our re-submission.

**2. Your current Financial Disclosure states, “OPP1120138”. However, your funding information on the submission form indicates that you received funding from “OPP119327”. Please indicate by return email the full and correct funding information for your study and confirm the order in which funding contributions should appear. Please be sure to indicate whether the funders played any role in the study design, data collection and analysis, decision to publish, or preparation of the manuscript.**

We apologise for this error. The correct funding code is OPP119327, with funding being received from the Bill and Melinda Gates Foundation. The funders played no roles in the study design, data collection and analysis, decision to publish, or preparation of the manuscript.

We have corrected the online financial disclosure statement so that it indicates OPP119327.

**3. In the online submission form, you indicated that "A de-identified dataset with variables included in this analysis can be made available on request to the Centre for Sexual Health & HIV/AIDS Research Zimbabwe, subject to ethical approval of a proposal.". All PLOS journals now require all data underlying the findings described in their manuscript to be freely available to other researchers, either 1. In a public repository, 2. Within the manuscript itself, or 3. Uploaded as supplementary information. This policy applies to all data except where public deposition would breach compliance with the protocol approved by your research ethics board. If your data cannot be made publicly available for ethical or legal reasons (e.g., public availability would compromise patient privacy), please explain your reasons by return email and your exemption request will be escalated to the editor for approval. Your exemption request will be handled independently and will not hold up the peer review process, but will need to be resolved should your manuscript be accepted for publication. One of the Editorial team will then be in touch if there are any issues.**

Should our manuscript be accepted for publication we will make the data available in a public repository. We are currently developing a de-identifiable dataset for this purpose.

**4. Please provide separate figure files in .tif or .eps format. For more information about figure files please see our guidelines.**

As requested, we have uploaded the figures in .tif format.

**5. Please insert an Ethics Statement at the beginning of your Methods section, under a subheading 'Ethics Statement'. It must include: 1) The name(s) of the Institutional Review Board(s) or Ethics Committee(s); 2) The approval number(s), or a statement that approval was granted by the named board(s); 3) (for human participants/donors) - A statement that formal consent was obtained (must state whether verbal/written) OR the reason consent was not obtained (e.g. anonymity). NOTE: If child participants, the statement must declare that formal consent was obtained from the parent/guardian.**

We now include an ethics statement and information on consent at the beginning of our methods section.

**Reviewer 1**

1. **Generally, the manuscript is well-written and organized. The study presents an important topic on recency testing and evaluates the use of DBS vs. Plasma for recency testing. The authors have described the study well in the introduction and its objective.** **Recommendation: Areas that need review by the authors are considered minor.**

We thank the reviewer for describing our submission as well written, organised, and covering an important topic.

2. **Line 65-66 (‘HIV 65 testing coverage has increased substantially and those attending services are likely to be representative of female sex workers (FSW) in Zimbabwe), the author’s statement highlighted in bold suggests that they are unsure of the population used in the study. Did you confirm that the enrolled participants were sex workers or were they persons seeking services from the mentioned clinics?**

The reviewer raises an interesting point. This sentence refers to the wide reach of the programme as it operated in 86 sites across all 10 provinces in Zimbabwe during the study period. CesHHARs routinely collected programme data indicates their services reach a large proportion of FSWs. The population served by the programme, and specifically in this study, are cisgender women and girls aged 18 years or older who specifically sell sex and self-identify as a sex worker. Analyses conducted by CeSHHAR have shown the women attending the programme to be very similar to the women selling sex participating in CeSHHAR Respondent Driven Sampling (RDS) studies. Although we are confident those attending our services will be representative of the entire FSW population in Zimbabwe, we prefer to position as “likely” as this reflects some uncertainty.

3. **The authors have used the Viral load test, HIV Lag, Avidity and ELISA test in determining the thresholds for either recent or long-term infection. However, the authors did not mention which ELISA kit was used, or the manufacturer’s name, similar to the HIV LAg Avidity test used in this study.**

In response to the reviewers comment we have amended the last paragraph of our methods to state we used an Enzygnost Anti-HIV 1/2 Plus ELISA test (Dade Behring, Inc., Marburg, Germany).

4. **The authors have not mentioned the sample input volume used in the testing for HIV Viral load, ELISA, and HIV LAg Avidity.**

We thank the reviewer for their comment. The sample input volumes applied were those described in each of the relevant assay Information-For-Use. For example, for the Lag avidity test, as according to the IFU, input volumes were 5ul for serum, and 100ul of a 6mm DBS specimen eluted in 500ul of elution buffer. In response to the reviewers comment we have included in our methods section the following sentence: “*All assays (including sample input volumes) were performed as per manufacturer’s instructions for use.*”.

5. **Could it be that the threshold used for HIV viral load testing using the DBS matrix contributed to the over classification of more recent infections compared to plasma Viral load?**

The reviewer raises an interesting point. We believe that this is unlikely as the comparability of DBS/Serum and Plasma specimens for viral load is well described. For example: <https://journals.asm.org/doi/10.1128/jcm.00418-11> and <https://www.sciencedirect.com/science/article/pii/S2667038022000667?via%3Dihub> and <https://pubmed.ncbi.nlm.nih.gov/26001044/>.

The cut -off applied for recency of 1000cpm is well described in the literature for use in a RITA for supporting the differentiating of recent from long- standing infection. Any variation around this cut-off would be minimal.

**Suggestion for your consideration:**

6. **See line 105-Can the authors consider presenting the data on the 58 recent infections in a 2by-2 table and have the sensitivity and specificity determined for each sample matrix?**

We thank the reviewer for their comment. We would argue that the use of sensitivity and specificity in this context is not appropriate as individuals can move between recent and long standing classification due to multiple confounding factors like initiation of treatment or viral rebound following treatment failure. To counter these factors the results are incorporated into a Recent infection Test Algorithm to determine classification. Even with the use of a RITA a false recency rate is possible. Having considered these issues, we decided to present our key results as shown in table 1.

7. **Could the results have been different if DBS were accorded similar storage treatment as plasma from collection to storage instead of having them stored at room temperature for 14 days? Could this have been a limitation in the design of this study?**

We thank the reviewer for their comment. The stability of DBS at room temperature for short periods of up to a month has been shown to have no effect on antibody measures. This advantage is one of the reasons we often use DBS in our programme testing. Longer periods of storage at room temperature could be problematic and storage of specimens at -80oC would then be advisable.
