# Supplementary material for: Comparing the test performance of dried-blood-spot and plasma HIV recent infection testing samples in a nationally scaled sex worker programme in Zimbabwe
Source: PLOS Glob Public Health. 2025 Nov 5;5(11):e0003791. doi: 10.1371/journal.pgph.0003791 (PMC12588490; doi:10.1371/journal.pgph.0003791)
Supplement: Attachment — Submitted filename: Rice_et_al_DBS_Response_to_2nd_round_reviewer_comments_auresp_3.docx [file pgph.0003791.s005.docx]

**26^th^ September 2025**

**Dear Editors,**

**Response to latest round of reviewer comments on our submitted research article – Comparing the test performance of dried-blood-spot and plasma HIV recent infection testing samples in a nationally scaled sex worker programme in Zimbabwe(PGPH-D-24-02067).**

We wish to thank the editors of PLOS Global Public Health for considering our submission for publication. We also wish to thank the editorial team and reviewers for their ongoing support and guidance. Below we present our response to each of the editorial and reviewer comments. Please note that these responses add to those provided in January of this year in relation to a previous round of editorial and reviewer comments and suggestions.

**Editorial comments**

1. **Details of sample processing and which sample used for which test are necessary.**

In response to comments, we have restructured and expanded our methods so that our sample processing is clearer. We present this information under the new sub-heading “Laboratory Procedures”.

2. **Subheadings might help**

In response to comments, we have inserted subheading in our methods section.

3. **Line 87: This statement starting with “Samples with a normalised optical density (ODn) <2.0…” seems very abrupt. A subheading or context may be necessary**

In response to this and the above comments we have restructured and expanded our methods.

4. **Line 107: “the authors state “of the 58 women with paired samples…” Where is this number coming from? Plasma identified 47 and DBS identified 57.**

In response to the reviewers comments we have amended our results to read “Fifty-eight women had a paired sample collection with a test result classified as recent. Of these women, 46 (79.3%) were concordant recent on both DBS and plasma, 11 (19%) were recent on DBS only, and 1 (1.7%) was recent on plasma only (table 1).

5. **Little or no statistical analyses details**

In response to the reviewers comment we have added a new sub-headed section to our methods.

**Reviewer 2 comments**

1. **What were the criteria for selecting women for paired sampling?**

In response to the reviewers question we have amended our methods under a new sub-header of “Study population and sample collection” to read “In addition to a DBS sample, ethylenediaminetetraacetic Acid (EDTA) whole blood samples were also collected from all consenting women at Sisters clinics pre-identified as having the capacity to transport collected samples to the laboratory within 24 hours.”. Our sentence now highlights how selection was at the clinic rather than the individual level.

2. **Were there systematic differences between the full cohort and the 464 paired-sample participants?**

We thank the reviewer for their question. In our sister publication (J Int AIDS Soc. 2024 Nov 26;27(12):e26391. doi: 10.1002/jia2.26391) we present detailed findings in relation to our full recency assay implementation, including a comparison of median age between all women testing HIV positive (30 years of age) and those enrolled in the study for recency testing (27 years of age). The purpose of our study here was to respond to persisting questions relating to potential differences in test results between plasma-based HIV recency testing and our standard approach using DBS. As such, our results (including both of our figures and our table) focus on the 464 women who were offered and consented to paired sample collection. Although we wish to remain focused on results pertaining to these 464 women, we will consider such a comparative analyse it if the reviewer feels it is essential.

3. **Clarify whether viral load was always measured from plasma or whether DBS VL was used in some cases. Variability here could affect classification outcomes.**

In response to the reviewers comment we now state the following under a new sub-header of “Laboratory Procedures”: “Viral load testing was subsequently performed with a NucliSens assay on all anti-HIV-1 positive DBS and plasma samples with a final ODn ≤1.5.”. All tests on plasma and DBS samples were performed and interpreted as per manufacturer’s instructions for use. We deliberate on classification outcomes in our discussion.

4. **Was the same ODn cut-off (1.5) applied to both DBS and plasma without adjustment?**

In response to the reviewers question we have amended our methods to read “All DBS and plasma samples with a final ODn >1.5 after retesting were classified as long-term HIV infection.”.

5. **No samples were reportedly rejected—this is uncommon. Please confirm how sample integrity and validity were assessed and whether any samples were excluded from analysis due to assay failure.**

We thank the reviewer for their interesting question. In this manuscript we present results from our 2021 to 2023 recency testing study in Zimbabwe. In 2018, we conducted the first implementation of recency testing in routine HIV testing settings in Kenya and Zimbabwe. During the implementation of three independent but linked studies of recency testing, we had a steep learning curve. For example, we repeatedly encountered invalid results from the LAg avidity assay and had to temporarily pause recruitment during month four of our 2018 study. To mitigate these challenges, we transferred testing to a back-up laboratory and, having compared operational procedures between the original and back-up laboratories, we were able to identify causes for the failed runs. The main reason for invalid results was found to be incorrect calibration and maintenance of the microplate washer. We report on the challenges we faced in our 2018 study, and the changes we implemented to overcome them, in a 2021 health services research publication (<https://doi.org/10.1186/s12913-021-06619-6>).

As informed by our 2018 studies, we put a lot of effort into collecting and storing specimens appropriately. As issues tend to arise when samples are stored inappropriately, all of our samples were collected and stored appropriately, and assay controls gave no cause for concern. Assay performance is also the focus of this study with us presenting difference between the matrices. In our discussion we highlight that “the use of different sample types impacted on recent HIV infection classification. On average, plasma samples had a higher ODn than DBS samples, with a mean difference of 0.53.” and that “In relation to false recency results, we were unable to explore this in our study beyond including a viral load in our RITA. However, we can assume that both the plasma and DBS recent classified results are likely to include misclassification.”.

6. **11 of 12 discordant cases were recent by DBS and long-standing by plasma—does this suggest DBS is systematically more sensitive, or perhaps more prone to false recency?**

The reviewer raises an important question. We report that, on average, plasma samples had a higher ODn than DBS samples which, as the reviewer highlights, resulted in a higher percentage of women being classified as recent positive on their DBS sample versus plasma. In our discussion we compare our results to the published literature (which is limited). We report how the authors of one study suggested “DBS protocols could be adjusted to account for false recency results, or that appropriate changes to assay cut-offs could be applied to account for the higher rate of recency positive test results arising from DBS.”. We go on to report that “In relation to false recency results, we were unable to explore this in our study beyond including a viral load in our RITA. However, we can assume that both the plasma and DBS recent classified results are likely to include misclassification.”. In our discussion we present results to support this assumption.

As the reviewer is probable aware, there are a lot of factors that could affect the performance of the DBS sample, including elution efficiency and haemtocrit levels. Given the information we have available, we fall on the side of DBS being more prone to false recency results than plasma. In our paper, however, we go on to discuss that “When conducting recency testing using DBS or plasma for different use-cases [2], there are factors in addition to test accuracy to be considered, for example implementation.”.

7. **Figure 1 and 2 are informative but would benefit from:**

- **Labelled axes with units and cut-off lines clearly indicated:** We have now added units to our axis labels and indicated the ≤1.5 cutoff: Plasma test ODn values (≤1.5 recency cut-off) and Dried blood spot test ODn values (≤1.5 recency cut-off).
- **A short caption describing key takeaways from the figure (e.g., “pink dots” are discordant):** We have added a footnote to the graph which states that test concordance is based on ≤1.5 cut-off. ----- Test concordance based on ≤1.5 ODn cut-off. Our legend now shows that discordant pairs are represented as pink dots.
- **Clearer legends differentiating recent/long-standing classifications:** We hope these have been addressed in the above two points.
- **Bland-Altman Plot: Consider adding limits of agreement and confidence intervals to better convey the precision of the ODn difference:** Although the plot we submitted had the limits of agreement (solid grey line) and the mean difference or bias (dashed line) shown, we are aware that we did not include a legend so have now made this clear. Including 95% CIs for non-horizontal LOA (our data do not meet the assumptions underlying horizontal LOA) we would argue is complicated and would not add much value to our analysis. Our aim was to assess whether the two methods agreed on recent HIV infection classification, and less about the statistical certainty.

8. **Include a summary of previous quantitative comparisons of ODn values between DBS and plasma (e.g., mean differences, variability) - Discuss whether the observed mean ODn difference (0.53) aligns with previous validation studies and manufacturer documentation.**

We thank the reviewer for their suggestion. In the first paragraph of our discussion we now include the sentence “On average, plasma samples had a higher ODn than DBS sample (mean difference of 0.53), with ODn agreement being better at values below 2”. We have also introduced a new paragraph (2^nd^ paragraph in our discussion) that compares our findings with those by Schlusser *et al*. As the reviewer is probably aware, the literature is limited in this area, hence our submission. We did consider results presented in the paper “Evaluation of an HIV recent infection testing algorithm with serological assays among men who have sex with men in Mexico” but refrained from including the findings in our discussion as they used mocked up DBS (like the schlusser paper but with a different level of red cells to mimic the hematocrit, so the variability you might see in hematocrit and elution efficiency may be different and therefore not a valid comparator).

9. **Cite examples of statistical methods used in the literature to adjust for these differences (e.g., regression calibration, cut-off optimization).**

Speaking to the reviewers’ comment, we highlight in our discussion how "DBS protocols could be adjusted to account for false recency results, or that appropriate changes to assay cut-offs could be applied to account for the higher rate of recency positive test results arising from DBS". There is a lack of evidence in this space, and exploring adjustment options was beyond the scope of this analysis. In response to the reviewers comment, we have added a new and final paragraph to our discussion which reads “When comparing DBS and plasma test results for the same assay, we need to consider potential differences in performance. We present information that will hopefully inform statistical adjustments to harmonise cut-offs on DBS and plasma assays, thereby improving the use and interpretation of recency assays in population-level HIV surveillance activities.”.

10. **Abstract: Consider stating the key numerical result from the ODn comparison (mean difference of 0.53) directly in the abstract.**

In response to the reviewers suggestion, we have amended our abstract accordingly.

11. **Clarify how confidentiality and anonymity were ensured given the sensitivity of the population (e.g., storage of consent forms, coded ID systems)**

In response to the reviewers comments we have expanded our methods to include “The programme has been described elsewhere [9]. In summary, all women are assigned a unique identification number on first contact with the programme, with collected data being held centrally in an anonymized DHIS2 database.” and “In addition to data collected routinely through the key population programme, anonymized enrolment details were collected and held on all participants in the central database, and were updated when RITA results were returned from the laboratory.”.

12. **Replace "dis-concordant" with the standard "discordant" throughout.**

We have amended our manuscript accordingly.

13. **Remove repeated sentences in the Methods section (lines 63–69 and 75–80 appear nearly duplicated).**

We have amended our manuscript accordingly.

14. **Table 1: Add row and column percent totals for better interpretability.**

We have amended our table accordingly.

15. **Ensure all figures and tables have explanatory footnotes or legends.**

We have amended our table and figures accordingly.

16. **Clearly state the objectives at the beginning of the section. For example, "This study aimed to assess the agreement between DBS and plasma samples for recent HIV infection testing and explore implications for misclassification and implementation."**

We thank the reviewer for their suggestion. At the end of our introduction we say “In the context of a national scaled sex worker programme in Zimbabwe we explored whether conducting plasma-based HIV recency testing, in addition to our standard approach using DBS, impacted on recent HIV infection classification.” and at the start of the discussion we have added “To gain additional insights into routine HIV recent infection testing within a national scaled sex worker programme in Zimbabwe, we assessed agreement between DBS and plasma samples and explored implications for misclassification and implementation.”.

17. **Elaborate on how a 2% discordance or the difference in ODn values could influence incidence estimation, public health decision-making, or resource allocation**.

In response to the reviewers comment we have added the following in relation to false recency test results arising from different assay performance in our discussions “Through reducing false recency test results, the inclusion of metabolite testing in a RITA can strengthen the strategic potential of these data to help us understand transmission dynamics and where interventions should be prioritised. However, we have previously highlighted how the inclusion of metabolite testing is costly and makes the implementation of a RITA more challenging.”.

18. **Provide an estimate of potential misclassification in your own dataset or clarify why it could not be estimated (e.g., lack of ART metabolite testing). Highlight this limitation explicitly.**

Whereas we have now added a few sentences on the use of ART metabolite testing, we prefer to highlight that “both the plasma and DBS recent classified results are likely to include misclassification” and support this statement with additional information than provide an estimate of potential misclassification. Our reason for this is that neither DBS or plasma can be see as a gold standard against which to measure the other. As highlighted by the reviewer, it is our primary aim to assess agreement between the DBS and plasma samples and explore implications for misclassification and implementation.

19. **Line 134–135: Consider rewording "a higher percentage of women being classified as recent positive" to "a higher percentage of women were classified as recently infected."**

We have amended our manuscript accordingly.

20. **Line 141–144: "We were unable to explore this..." would benefit from clearer justification. Could mention logistical or ethical constraints.**

In relation to the reviewers comment we have added to our discussion “However, we have previously highlighted how the inclusion of metabolite testing is costly and makes the implementation of a RITA more challenging [4].”.

21. **Line 151: "Preferably in combination with other complementary data" is vague. What type of data is meant here?**

As we now talk about metabolite data elsewhere, and agree this statement is vague and adds little, we have removed this sentence.

22. **Lines 157–160: Good point about storage, but could be stronger if supported with cost or logistical data.**

We agree with the reviewer that our statement would be stronger if supported with cost or logistical data. We had hoped to collect additional logistics data through the use of cool box trackers. Additional implementation challenges arising in relation to the COVID pandemic resulted in these data not being collected.

23. **Line 167: Consider citing specific limitations of point-of-care tests mentioned in references [14,15].**

In response to the reviewers suggestion, we now focus on the Ugandan study as it aligns with our research focusing on validating recency assays for real world use. At the end of our discussion, we now provide further details on the Ugandan study findings.

**Reviewer 3 comments**

1. **My major comment turns around the validation of the test used. Usually, when comparing two methods or two samples, there should be a gold standard, something to validate the results. The authors should not rely on the test performances provided by the manufacturer, but should have made a prior validation of the test used and the best example would be to use sample from individuals with known time of infection.**

We thank the reviewer for their comments. Having presented our 2018 recency study results to groups such as the WHO team responsible for assay prequalification and the Forum for Collaborative Research Recency Assay Working Group, an interest in assessing agreement between DBS and plasma samples and exploring implications for misclassification and implementation in real world settings was expressed. As this was the aim of our 2021-23 study, we apply our assays to samples collected as part of routine programme delivery in Zimbabwe where time of infection among those testing positive for HIV is not known. In presenting our results, we hope to improve the use and interpretation of recency assays not only in programme delivery but also in population-level HIV surveillance activities. Although we had no gold standard against which to compare (a reality in routine service delivery), we have amended our discussion to include results from a study in Uganda where recency point of care test results were validated against a gold-standard.

2. **While 47 women were found to be recently infected using plasma, there were 57, when the authors used whole DBS. The difference is high and no one can tell for sure if these 10 individuals were recently infected or not. It may turn out that this difference is partly the consequence of the overall sensitivity and/or specificity of the test used.**

The reviewer refers to our key aim and results where we highlight differences in the percentage of women classified as recent by DBS (12.3%; 57/464) and plasma (10.1%; 47/464). In response to reviewer comments we have our expanded our discussion to compare our results to those from other studies. We now suggest the differences are likely due to misclassification / false recency test results. We also now highlight how the inclusion of viral load test results and, where possible, antiretroviral therapy metabolite test results, may reduce false recency test results.

3. **In addition, results are a bit confusing; how was the 97% agreement obtained?**

In response to the reviewers comments we have emboldened the discordant samples in table 1 and added a footnote to clearly distinguish between agreement and disagreement. We have also amended the sentence prior to table 1 to read “Overall, there was 97.4% (452/464) concordance between the DBS and plasma samples (in addition to the 46 women concordant recent, 406 women were concordant long-standing).”.

4. **The authors mentioned 58 women with paired samples who had result classified as recent….where this number of 58 from?**

In response to the reviewers comment we have amended our results to read “Fifty-eight women had a paired sample collection with a test result classified as recent. Of these women, 46 (79.3%) were concordant recent on both DBS and plasma, 11 (19%) were recent on DBS only, and 1 (1.7%) was recent on plasma only (table 1).”.

5. **I tough there were 464 individuals who provided the two biology specimen ….. and again the concordance in this 58 women is different from the initial 97% concordance. The authors should please clarify the results.**

The reviewer is correct in highlighting that 464 women were offered and consented to paired sample collection, that there was overall agreement of 97% between DBS and plasma, and that 58 women overall were classified as recent either on both samples or only one of the two samples. In response to the reviewers comments we have amended our results to read as follows “Fifty-eight women had a paired sample collection with a test result classified as recent. Of these women, 46 (79.3%) were concordant recent on both DBS and plasma, 11 (19%) were recent on DBS only, and 1 (1.7%) was recent on plasma only (table 1). Eleven samples were deemed long-standing by the plasma assay but recent by the paired DBS, and one deemed long-standing by DBS but recent by the paired plasma sample. Overall, there was 97.4% (452/464) concordance between the DBS and plasma samples (in addition to the 46 women concordant recent, 406 women were concordant long-standing).”.
