# Supplementary material for: Comparing the test performance of dried-blood-spot and plasma HIV recent infection testing samples in a nationally scaled sex worker programme in Zimbabwe
Source: PLOS Glob Public Health. 2025 Nov 5;5(11):e0003791. doi: 10.1371/journal.pgph.0003791 (PMC12588490; doi:10.1371/journal.pgph.0003791)
Supplement: Attachment — Submitted filename: Rice et al_DBS_Response to 2nd round reviewer comments.docx [file pgph.0003791.s004.docx]

**2^nd^ July 2025**

**Dear Editors,**

**Response to latest round of reviewer comments on a submitted research article – Comparing the test performance of dried-blood-spot and plasma HIV recent infection testing samples in a nationally scaled sex worker programme in Zimbabwe(PGPH-D-24-02067).**

We wish to thank the editors of PLOS Global Public Health for considering our submission for publication. We also wish to thank the reviewer for their ongoing support and guidance. Below we provide detail of our response to each of the reviewer comments.

**Editorial comments**

1. **Details of sample processing and which sample used for which test are necessary.**

In response to the reviewers’ comments, we have restructured and expanded our methods so that our sample processing is clear. We present this information under the new sub-heading “Laboratory Procedures”.

2. **Subheadings might help**

In response to the reviewers comments we have inserted subheading in our methods section.

3. **Line 87: This statement starting with “Samples with a normalised optical density (ODn) <2.0…” seems very abrupt. A subheading or context may be necessary**

In response to this and the above comments we have restructured and slightly expanded our methods

4. **Line 107: “the authors state “of the 58 women with paired samples…” Where is this number coming from? Plasma identified 47 and DBS identified 57.**

In response to the reviewers comments we have amended our results to read “Fifty-eight women had a paired sample collection with a test result classified as recent. Of these women, 46 (79.3%) were concordant recent on both DBS and plasma, 11 (19%) were recent on DBS only, and 1 (1.7%) was recent on plasma only (table 1).”.

5. **Little or no statistical analyses details**

In response to the reviewers comment we have added a new sub-headed section to our methods.
